# Radial Perforation: Rescue With Internal Tamponade

**DOI:** 10.1016/j.jaccas.2025.106336

**Published:** 2025-12-03

**Authors:** Matthew D. Hordern, Sarah L. Fairley, Scott A. Harding

**Affiliations:** Cardiology Department, Wellington Hospital, Wellington, New Zealand

**Keywords:** percutaneous intervention, perforation, radial, ST-segment elevation myocardial infarction

## Abstract

Radial artery perforation may result in hematoma and, in rare cases, compartment syndrome with increased risk of this complication in the setting of multiple antithrombotic agents. Radial artery perforation is usually managed by external compression and reversal of anticoagulation. In this clinical vignette, we report management of a radial artery perforation in the setting of rescue percutaneous coronary intervention with internal tamponade using a sheathless guiding catheter. This represents a simpler strategy than external compression and allows completion of the coronary procedure while treating the perforation.

**Take-Home Messages:**

Radial artery perforation can safely and effectively be managed with internal tamponade using a sheathless guiding catheter rather than external compression.

## Graphical Abstract

**Figure:**
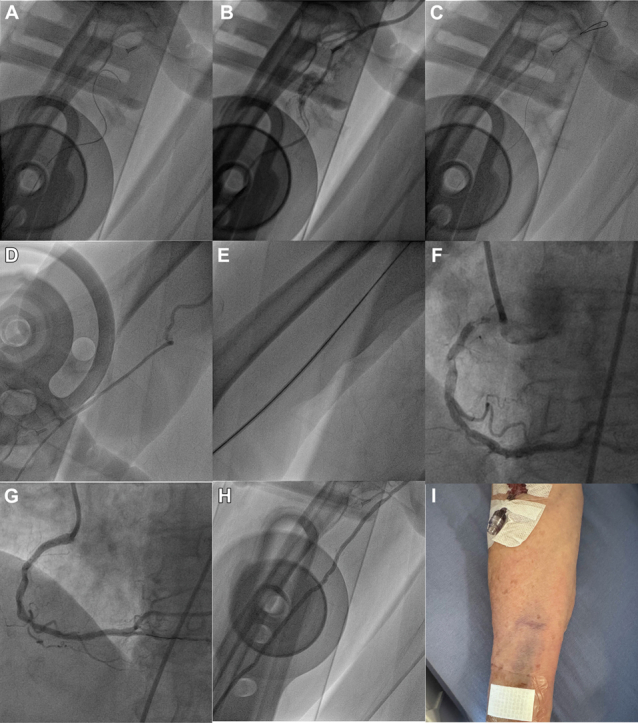

A 77-year-old female was thrombolysed with tenecteplase (17.5 mg) for an ST-segment elevation myocardial infarction and transported to our hospital. She received aspirin (300 mg), Clopidogrel (75 mg), and clexane (50 mg).^1^

Due to ongoing pain and inferolateral ST-segment elevation, the patient was taken directly for cardiac catheterization. Right radial access was established using a 6-F Glidesheath Slender (Terumo). Attempts to pass a 0.035-inch 0.5-cm J wire and then a 0.035-inch Glidewire (Figure 1A, Video 1) were unsuccessful. A radial angiogram demonstrated spasm, dissection, and perforation of the radial artery which was tortuous and had an aberrant origin form the upper arm (Figure 1B, Video 2).

**Figure 1:**
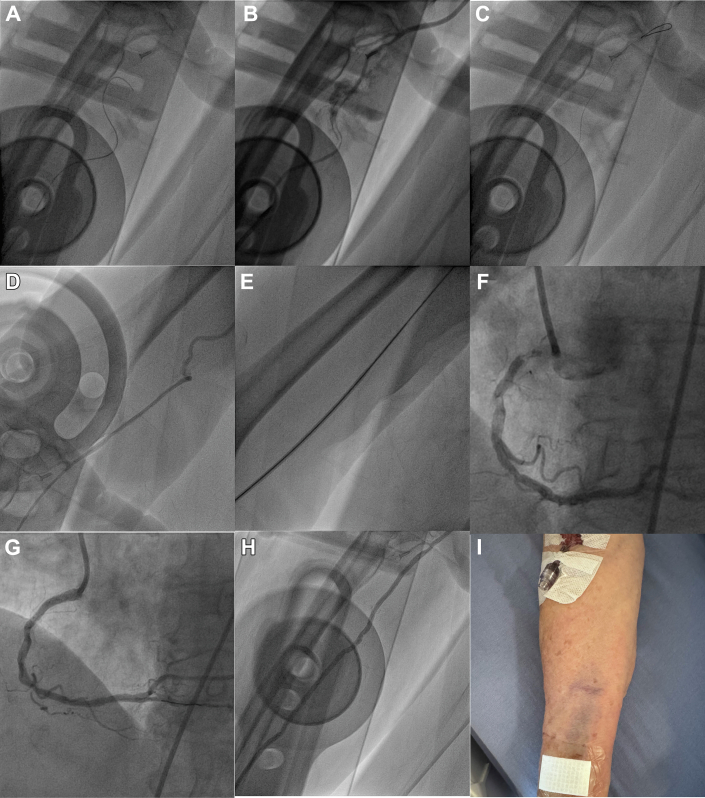
Radial Perforation Managed With Internal Tamponade (A) Failure to advance a 0.035-inch glidewire up the radial artery. (B) Radial angiogram demonstrating radial spasm, dissection, and perforation. (C) Successful advancement of a knuckled coronary 0.014-inch guidewire in the radial artery. (D) Angiogram in the upper arm demonstrating a high origin and tortuosity of the radial artery. (E) The Sheathless Eaucath Guiding Catheter (SEGC) passes easily through the radial artery. (F) Coronary angiography demonstrating a severe calcific ostial right coronary artery (RCA) lesion. (G) Coronary angiography following percutaneous coronary intervention of the ostial RCA lesion. (H) Angiography of the radial artery following removal of the SEGC demonstrating resolution of the dissection and perforation. (I) Photograph of the right forearm the morning following the procedure with minor bruising at the puncture site but no forearm hematoma.

An attempt to cross the dissected radial artery in the true lumen using a Sion Blue coronary wire (Seto) was unsuccessful. A mini knuckle enabled it to be advanced easily in the true lumen without resistance (Figure 1C, Video 3). A 4-F RBR catheter (Merit) was advanced over the Sion Blue wire, and an angiogram was performed (Figure 1D). A 6.5-F JR4 Sheathless Eaucath Guiding Catheter (SEGC) (Seto) was then exchanged over a 0.035-inch guide wire. The SEGC passed through the radial artery (Figure 1E, Video 4) and tortuous brachiocephalic into the aorta without difficulty. Coronary angiography was performed and demonstrated a calcified ostial right coronary artery subtotal occlusion. Due to a lack of support via radial access from tortuosity, femoral access was gained. The SEGC was left in place in the radial artery while the intervention to the right coronary artery was completed via the femoral artery. Following completion of the case, the SEGC was withdrawn, and angiography performed demonstrating resolution of the dissection and perforation (Figure 1H, Video 5).

Radial perforation in the setting of rescue percutaneous coronary intervention poses high risk of forearm hematoma development and possible compartment syndrome due to multiple antithrombotic agents.^2^ While radial perforation can be managed with external compression, this is not reliable and is poorly tolerated. An alternative strategy is internal tamponade, which can be provided by a guide catheter and has the advantage of allowing completion of the percutaneous coronary intervention. This strategy relies on passage of a guidewire in the true lumen. A coronary guidewire with a low tip load often facilitates crossing. Formation of a knuckle may be helpful in avoiding side branches. Care must be taken to advance the knuckled coronary guidewire and ensure there is no resistance. The SEGC has a tapered central dilator to aid introduction over a 0.035-inch guidewire. The tapered shape of the dilator and absence of a gap between the tip of the dilator and the 0.035-inch guidewire avoid the so-called “razor effect” that occurs when passing a standard guide catheter. This along with the hydrophilic coating are features that aid delivery through areas arterial spasm or injury.^3^

The patient made an uncomplicated recovery without evidence of forearm hematoma or requiring forearm compression (Figure 1I). A follow-up radial ultrasound demonstrated the radial artery was patent with no obvious sign of injury (Videos 6 and 7, Supplementary Figures 1 and 2).

## Funding Support and Author Disclosures

Dr Harding has received honoraria for proctoring and speaking for Abbott Vascular, Boston Scientific, Teleflex Medical, and Bio-Excel; and research funding from Asahi Intecc and Reflow Medical. All other authors have reported that they have no relationships relevant to the contents of this paper to disclose.

## Appendix

Video 1 Unsuccessful Attempts to Pass a 0.035-inch Glidewire Through the Radial Artery

Video 2 Radial Angiogram Demonstrating Spasm, Dissection, and Perforation of the Radial Artery

Video 3 Using a Mini Knuckle to Advance a Sion Blue Wire Easily in the True Lumen Without Any Resistance

Video 4 The Sheathless Eaucath Guiding Catheter Passed Through the Radial Artery and Tortuous Brachiocephalic Into the Aorta Without Difficulty

Video 5 Following Completion of the Percutaneous Coronary Intervention, the Sheathless Eaucath Guiding Catheter was Withdrawn and Angiography Performed Demonstrating Resolution of the Dissection and Perforation

Video 6 Follow-Up Cross Sectional Ultrasound of Radial Artery

Video 7 Follow-Up Longitudinal Ultrasound of Radial Artery

Supplemental Figures 1 and 2 Supplementary Figure 1: Follow-Up Cross Sectional Ultrasound of Radial Artery Supplementary Figure 2: Follow-Up Longitudinal Ultrasound of Radial Artery
