# Supplementary material for: Radial Perforation: Rescue With Internal Tamponade
Source: JACC Case Rep. 2025 Dec 3;31(6):106336. doi: 10.1016/j.jaccas.2025.106336 (PMC12905710; doi:10.1016/j.jaccas.2025.106336)

**Supplementary Figure 1.** Follow-Up Cross Sectional Ultrasound of Radial Artery


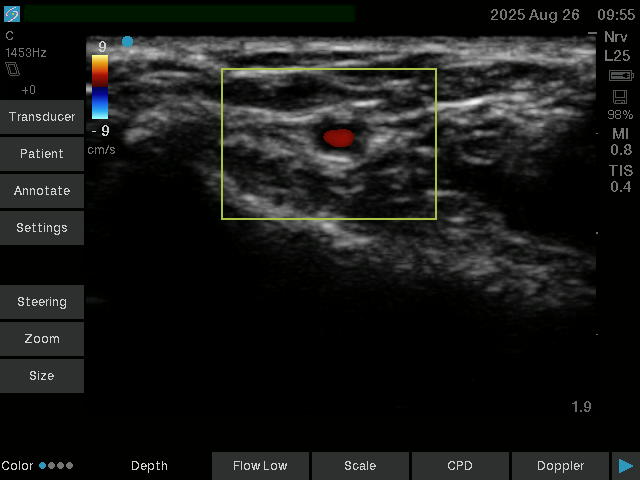


**Supplementary Figure 2.** Follow-Up Longitudinal Ultrasound of Radial Artery


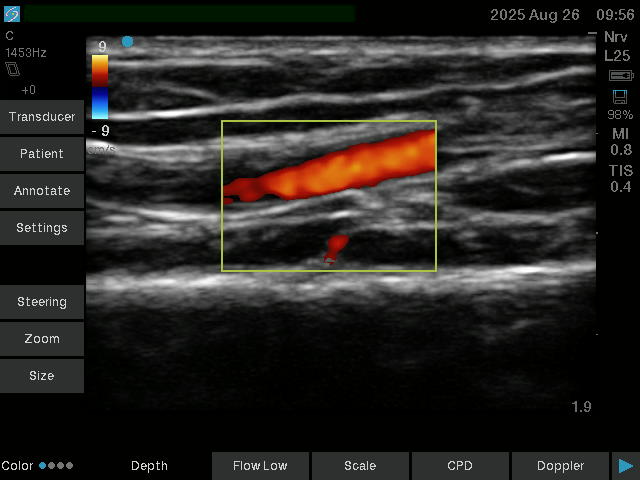

Supplement: Supplemental Figures 1 and 2 — Supplementary Figure 1: Follow-Up Cross Sectional Ultrasound of Radial Artery Supplementary Figure 2: Follow-Up Longitudinal Ultrasound of Radial Artery [file mmc8.docx]
